# Saving 500 Lives Campaign: another way to improve the mortality rate of patients with severe sepsis and septic shock

**DOI:** 10.1186/cc11792

**Published:** 2012-11-14

**Authors:** R Champunot, N Kamsawang, P Tuandoung, S Tansuphaswasdikul

**Affiliations:** 1 Buddhachinaraj Phitsanulok Hospital, Phitsanulok, Thailand

## Background

In September 2010, 9 months after empowerment of caring for patients with severe sepsis and septic shock and implementation of a cooperative sepsis management protocol between community hospitals and tertiary referral hospital in Phitsanulok, the Phitsanulok Co-operative Sepsis Management (PCSM) team announced the Saving 500 Lives Campaign. This campaign aimed to encourage the unity and importance of caring for patients with severe sepsis and septic shock.

## Methods

**Figure 1:**
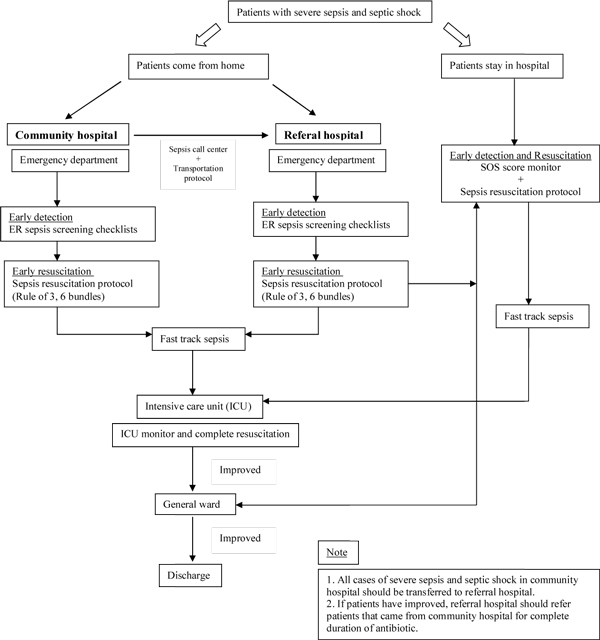
**Severe sepsis and septic shock strategy in Phitsanulok, Thailand**.

From October 2010 to September 2011, eight community hospitals and one tertiary referral hospital in Phitsanulok established and promoted a set of achievable goals and interventions for patients with severe sepsis and septic shock (Figure 1). These interventions included: establishing sepsis team in all hospitals to coordinate and monitor the achievement goals; using a search out severity (SOS) score and a sepsis screening tool to help early and more accurate diagnosis; providing early resuscitation protocol and other measures as indicated by implementing a checklist for sepsis management (sepsis six bundles, rule of three, early goal-directed therapy); communication and providing important information during transportation using a protocol; and early intensivist involvement and rapid transfer to the ICU from the emergency department using a sepsis fast-track protocol. Physician and nurse leadership actively engaged in the data review and shared ideas in every hospital for improvement and closely tracked progress against those goals and interventions. The mortality rate of patients with severe sepsis and septic shock was used to measure the success of the campaign.

## Results

The Saving 500 Lives Campaign succeeded in efforts to saved 660 patients from 1,048 patients with severe sepsis and septic shock in Phitsanulok, Thailand. The total mortality rate was 37% (decreased from 47% in the past year). The group of patients from community hospitals that can be admitted directly to the ICU from the emergency department using a sepsis fast-track protocol had the lowest mortality rate (19%).

## Conclusion

Setting a numeric goal using the 500 Lives Campaign was another way to support and empower the cooperation of sepsis care in Phitsanulok, Thailand.
